# Machine Learning Approach for Classifying Multiple Sclerosis Courses by Combining Clinical Data with Lesion Loads and Magnetic Resonance Metabolic Features

**DOI:** 10.3389/fnins.2017.00398

**Published:** 2017-07-11

**Authors:** Adrian Ion-Mărgineanu, Gabriel Kocevar, Claudio Stamile, Diana M. Sima, Françoise Durand-Dubief, Sabine Van Huffel, Dominique Sappey-Marinier

**Affiliations:** ^1^CREATIS Centre National de la Recherche Scientifique UMR5220 & Institut National de la Santé et de la Recherche Médicale, U1206, Université de Lyon, Université Claude Bernard-Lyon 1, INSA-Lyon Villeurbanne, France; ^2^Department of Electrical Engineering (ESAT), STADIUS Center for Dynamical Systems, Signal Processing and Data Analytics, KU Leuven Leuven, Belgium; ^3^imec Leuven, Belgium; ^4^R&D Department, icometrix Leuven, Belgium; ^5^Service de Neurologie A, Hôpital Neurologique, Hospices Civils de Lyon Bron, France; ^6^CERMEP - Imagerie du Vivant, Université de Lyon Bron, France

**Keywords:** multiple sclerosis, longitudinal analysis, magnetic resonance spectroscopic imaging, EDSS, lesion load, machine learning

## Abstract

**Purpose:** The purpose of this study is classifying multiple sclerosis (MS) patients in the four clinical forms as defined by the McDonald criteria using machine learning algorithms trained on clinical data combined with lesion loads and magnetic resonance metabolic features.

**Materials and Methods:** Eighty-seven MS patients [12 Clinically Isolated Syndrome (CIS), 30 Relapse Remitting (RR), 17 Primary Progressive (PP), and 28 Secondary Progressive (SP)] and 18 healthy controls were included in this study. Longitudinal data available for each MS patient included clinical (e.g., age, disease duration, Expanded Disability Status Scale), conventional magnetic resonance imaging and spectroscopic imaging. We extract *N*-acetyl-aspartate (NAA), Choline (Cho), and Creatine (Cre) concentrations, and we compute three features for each spectroscopic grid by averaging metabolite ratios (NAA/Cho, NAA/Cre, Cho/Cre) over good quality voxels. We built linear mixed-effects models to test for statistically significant differences between MS forms. We test nine binary classification tasks on clinical data, lesion loads, and metabolic features, using a leave-one-patient-out cross-validation method based on 100 random patient-based bootstrap selections. We compute F1-scores and BAR values after tuning Linear Discriminant Analysis (LDA), Support Vector Machines with gaussian kernel (SVM-rbf), and Random Forests.

**Results:** Statistically significant differences were found between the disease starting points of each MS form using four different response variables: Lesion Load, NAA/Cre, NAA/Cho, and Cho/Cre ratios. Training SVM-rbf on clinical and lesion loads yields F1-scores of 71–72% for CIS vs. RR and CIS vs. RR+SP, respectively. For RR vs. PP we obtained good classification results (maximum F1-score of 85%) after training LDA on clinical and metabolic features, while for RR vs. SP we obtained slightly higher classification results (maximum F1-score of 87%) after training LDA and SVM-rbf on clinical, lesion loads and metabolic features.

**Conclusions:** Our results suggest that metabolic features are better at differentiating between relapsing-remitting and primary progressive forms, while lesion loads are better at differentiating between relapsing-remitting and secondary progressive forms. Therefore, combining clinical data with magnetic resonance lesion loads and metabolic features can improve the discrimination between relapsing-remitting and progressive forms.

## 1. Introduction

Multiple sclerosis (MS) is an inflammatory disorder of the brain and spinal cord in which focal lymphocytic infiltration leads to damage of myelin and axons (Compston and Coles, 2008). MS affects approximately 2.5 million people worldwide, with an onset age commonly between 20 and 40 years, and an incidence more than twice as high in women compared to men (McAlpine and Compston, 2005).

The majority of MS patients (85%) usually experience a first attack defined as Clinically Isolated Syndrome (CIS), and will develop a relapsing-remitting (RR) form (Miller et al., 2012). Two thirds of the RR patients will develop a secondary progressive (SP) form, while the other third will follow a benign course (Scalfari et al., 2010). The rest of MS patients (15%) will start directly with a primary progressive (PP) form.

The criteria to diagnose MS forms was originally formulated by McDonald et al. (2001) and revised by Polman et al. (2005, 2011). They all rely on using conventional magnetic resonance imaging techniques (MRI) such as T1-weighted, gadolinium-enhanced T1-weighted MRI, as well as T2-weighted and FLAIR, due to a high sensitivity for visualizing MS lesions. Conventional MRI is also used for quantifying lesion load (LL), a marker of inflammation process but only a moderate predictor of MS evolution (Filippi et al., 1994).

More recently, advanced magnetic resonance techniques such as ^1^H-Magnetic Resonance Spectroscopic Imaging (MRSI), Diffusion Tensor Imaging (DTI), and Magnetization Transfer Imaging (MTI) have been shown (Rovira et al., 2013) to provide a better characterization of the normal appearing white matter (NAWM) and thus a better understanding of the pathological mechanisms of MS. MTI metrics reflect the demyelination and remyelination processes and have been shown to predict the evolution of MS lesions. DTI metrics are very sensitive to the MS pathology and have been shown to be mainly affected by myelin loss and decreased neuronal integrity. MRS metrics provide high MS pathological specificity as well as high sensitivity to biochemical changes. Decrease of *N*-acetyl-aspartate (NAA) was observed in both chronic lesions and NAWM, reflecting a neuronal integrity loss (Rovira et al., 2013). Choline (Cho) and Creatine (Cre) contents were found to be increased in WM lesions and in NAWM, indicating the presence of severe demyelination and cell proliferation in relation with inflammatory processes (Tartaglia et al., 2002; Sajja et al., 2009).

Therefore, in this study we investigate the added value of magnetic resonance metabolic features (NAA/Cho, NAA/Cre, Cho/Cre) combined with routinely collected clinical MS data [e.g., patient age, disease duration (DD), Expanded Disability Status Scale (EDSS)] and lesion load values (LL). To this purpose, we build multiple binary classifiers to automatically discriminate between different clinical forms of MS patients, by training each classifier on combinations of clinical data, lesion loads, and metabolic features.

## 2. Materials and methods

### 2.1. Patient population

Eighty-seven MS patients (12 CIS, 30 RR, 28 SP, and 17 PP) were included in this study, while 18 volunteers without any neurological disorders served as healthy control (HC) subjects. Diagnosis and disease course were established according to the McDonald criteria (Lublin and Reingold, 1996; McDonald et al., 2001), while disability was assessed with EDSS. This prospective study was approved by the local ethics committee (CPP Sud-Est IV) and the French national agency for medicine and health products safety (ANSM) and written informed consents were obtained from all patients and control subjects prior to study initiation. More details for each MS group, such as average age at first scan, average disease duration, median EDSS, and average lesion loads can be found in Table 1.

**Table 1:** Patient population: Age − average value (standard deviation); Disease duration − average value (standard deviation); EDSS − median (minimum − maximum); Lesion Load − average value (standard deviation).

	**CIS**	**RR**	**PP**	**SP**
Number of patients (Male/Female)	12 (6/6)	30 (6/24)	17 (6/11)	28 (17/11)
Age at first scan (years)	31.8 (6.4)	33.2 (7)	39.5 (6)	41.1 (4.8)
Disease duration (years)	2.9 (1.9)	8.3 (4.8)	7.5 (2.9)	14.9 (6.1)
EDSS median (range)	1 (0–4)	2 (0–5.5)	4 (2–7.5)	5 (3–8.5)
Lesion Load (ml)	6.6 (3.5)	16.7 (12.6)	20.8 (13)	31 (12.9)
Total number of scans	62	226	125	206

### 2.2. Longitudinal MS data

The MS patients involved in this study were scanned multiple times over a different period for each patient, ranging from 2.5 to 6 years. The minimum number of scans is 3, while the maximum is 10. The gap between two consecutive scans is either 6 months or 1 year. In total there are 619 MS scans, but because of missing lesion loads and metabolic features, there are 592 (95.6%) scans with full complete data, leading to an average of 6–7 complete scans/patient.

### 2.3. MRI acquisition and processing

All patients and control subjects underwent MR examination using a 1.5 Tesla MR system (Sonata Siemens, Erlangen, Germany) and an 8 elements phased-array head-coil.

#### 2.3.1. Conventional MRI

Conventional MRI protocol consisted of a 3 dimensional T1-weighted (magnetization prepared rapid gradient echo-MPRAGE) sequence with repetition time/echo time/time for inversion (TR/TE/TI) = 1, 970/3.93/1, 100 ms, flip angle = 15°, matrix size = 256 × 256, field of view (FOV) = 256 × 256 mm, slice thickness = 1 mm, voxel size = 1 × 1 × 1 mm, acquisition time = 4.62 min, and a fluid attenuated inversion recovery (FLAIR) sequence with TR/TE/TI = 8, 000/105/2, 200 ms, flip angle = 150°, matrix size = 192 × 256, field of view (FOV) = 240 × 240 mm, slice thickness = 3 mm, voxel size = 0.9 × 0.9 × 3 mm, acquisition time = 4.57 min.

#### 2.3.2. MRSI acquisition

MRSI data was acquired from one slice of 1.5 cm thickness, placed above the corpus callosum and along the anterior commissure - posterior commissure (AC-PC) axis, encompassing the centrum semioval region, and took 5 min and 20 s. A point-resolved spectroscopic sequence (PRESS) with TR = 1,690 ms and TE = 135 ms was used to select a volume of interest (VOI) of 105 × 105 × 15 mm^3^ during the acquisition of 24 × 24 (interpolated to 32 × 32) phase-encodings over a field of view (FOV) of 240 × 240 mm^2^.

#### 2.3.3. MRSI processing

MRSI data processing was performed using SPID (Poullet, 2008; Poullet et al., 2008) in MatLab 2015a (MathWorks, Natick, MA, USA). AQSES-MRSI (Poullet et al., 2007; Sava et al., 2011) was used to quantify *N*-acetyl-aspartate, Choline (Cho), and Creatine (Cre), using a synthetic basis set. The basis set incorporates prior knowledge of the individual metabolites in the quantification procedure. MPFIR (maximum-phase finite impulse response) filtering (Sundin et al., 1999) was included in the AQSES-MRSI procedure for residual water suppression, with a filter length of 50 and spectral range from 1.9 to 3.4 ppm. A band of two voxels at the outer edges of each VOI was discarded in order to avoid chemical shift displacement artifacts and lipid contamination artifacts.

#### 2.3.4. Quality control

After quantifying metabolites from all MRSI grids, a quality control was performed. Voxels with Cramer-Rao Lower Bounds (CRLBs) lower than 10% for *each* of the three metabolites (NAA, Cho, and Cre) were kept as having “good quality” to perform feature extraction. If the number of “good quality” voxels is lower than 50% of the total amount of voxels in the MRSI grid, then the acquisition is discarded. All 18 Control subjects had MRSI data with a number of “good quality” voxels higher than 50% of the total amount of voxels, and 606 out of 619 (97.9%) MRSI data from MS patients had good quality as defined earlier.

### 2.4. Feature extraction

In this study we use three types of features: clinical (e.g., patient age, disease duration, and EDSS), lesion loads, and metabolic features. The clinical features are routinely acquired in the hospital. The lesion loads were computed based on T1 and FLAIR, using the MSmetrix software (Jain et al., 2015) developed by icometrix (Leuven, Belgium). The computation of metabolic features was performed in two steps: three metabolic ratios (NAA/Cho, NAA/Cre, Cho/Cre) were computed for each “good quality” voxel and then averaged, leading to three metabolic features extracted from each MRSI grid.

### 2.5. Training approach

Nine binary classification tasks were studied: HC vs. CIS, HC vs. RR, HC vs. PP, HC vs. RR+SP, HC vs. PP+SP, CIS vs. RR, CIS vs. RR+SP, RR vs. PP, RR vs. SP. The first three tasks investigated differences between HC and the starting MS forms (CIS, RR, and PP). The next task investigated differences between HC and MS patients that are likely to evolve or had evolved into secondary progressive form (RR+SP). Afterwards, we investigated differences between HC and definite progressive forms (PP+SP). The next two tasks investigated differences between CIS patients and the most likely progression of CIS, namely RR and RR+SP. From a neurological point of view, the last two tasks were the most intriguing, as they were discriminating between the most common inflammatory MS form (RR) and the two progressive forms, PP and SP.

For each task, data normalization was performed. We use a leave-one-patient-out cross-validation (LOPOCV) setup, meaning that all data points of each patient will be in the test set, and will be classified based on a model learned on a training set with *n*−1 data points corresponding to *n*−1 patients, where *n* is the total number of patients, different for each classification task (e.g., for HC vs. CIS, *n* = 30). Because each patient has at least 3 data points, we randomly select one data point to be in the training set. We repeat the random sampling for each patient in the training set, and repeat the whole procedure 100 times. Therefore, each data point will be assigned 100 times to either class 1 or class 2, and in the end it will be assigned to one of the classes according to majority voting. This procedure is repeated until all patients from each classification task have been tested.

By using this random patient-based bootstrap sampling, the two classes in the training set have a more balanced distribution of points (18 HC, 12 CIS, 30 RR, 17 PP, 28 SP), compared to using the total number of points of each class (18 HC, 61 CIS, 214 RR, 121 PP, 196 SP).

### 2.6. Performance measures and statistical testing

For each task, we computed and reported four measures, in percentage: F1-score, sensitivity, specificity, and balanced accuracy rate (BAR). We explain these four measures using the general confusion matrix in Table 2.

**Table 2:** General confusion matrix.

**Confusion matrix**		**Predicted condition**	
		**Predicted negative**	**Predicted positive**
True condition	Condition negative	True negative (TN)	False positive (FP)
	Condition positive	False negative (FN)	True positive (TP)

The four measures are defined by the following formulas: $F1=\frac{2\times TP}{2\times TP+FN+FP}$, $Sensitivity=\frac{TP}{TP+FN}$, $Specificity=\frac{TN}{TN+FP}$, $BAR=\frac{Sensitivity+Specificity}{2}$.

Throughout our study the positive class was the first class from each of the nine binary classification tasks: HC for the first 5 tasks, CIS for the 6th and 7th tasks, and RR for 8th and 9th tasks.

In order to correctly assess if there are significant differences between the four MS groups, we built several linear mixed effects models which were able to incorporate the temporal evolution of each patient's MS course. We used five fixed effects and two random effects. The fixed effects are: MS course, gender, disease onset age, disease duration, and the interaction between MS course and disease duration. The random effects are set for each patient allowing an individual starting point and an individual disease evolution. The most interesting fixed effect for this study is the first one, which represents the average of the response variable at the beginning of the MS course, or when “disease duration” = 0. We built four linear mixed effects models, one for each response variable: NAA/Cho, NAA/Cre, Cho/Cre, and lesion load. All statistical models were built in the “R” software using the “lme4” package (Bates, 2010), statistical testing was done using the “lmerTest” package (Kuznetsova et al., 2015) and *post-hoc* analysis was done using the “multcomp” package (Hothorn et al., 2008). All tests were done for a significance level (α) of 0.05.

### 2.7. Classifiers

Three supervised classifiers implemented in Python 2.7.11 with scikit-learn 0.17.1 (Pedregosa et al., 2011) have been used: Linear Discriminant Analysis (LDA), Support Vector Machines (SVM), and Random Forest (RF). We tuned each classifier's parameters by optimizing the F1-score over a five-fold cross validation on the training set within a grid search of individual parameters, specified further for each particular classifier. Fisher's LDA (Fisher, 1936) is based on a linear combination of input features, with three possible solvers: singular value decomposition, least squares solution, and eigenvalue decomposition. Tuning involved choosing between the first solver and the last two solvers combined with shrinkage varying from 0 to 1 in steps of 0.1. Class unbalance was adjusted by setting the *priors* parameter equal to class probabilities. We use SVM (Cortes and Vapnik, 1995; Cristianini and Shawe-Taylor, 2000) with a radial basis function kernel (SVM-rbf), defined by two parameters: C, or the misclassification cost, and γ, which is proportional to the inverse of a support vector's radius of influence. We tuned C and γ by performing a logarithmic grid search between 0.00001 and 100,000. Class unbalance was adjusted by setting the *class_weight* parameter to *balanced*. Random Forests (Breiman, 2001) is based on a group of decision trees. We tune the number of decision trees between 200, 400, 600, 800, and 1,000. Class unbalance was adjusted by setting the *class_weight* parameter to *balanced_subsample*.

## 3. Results

**Figure 1:**
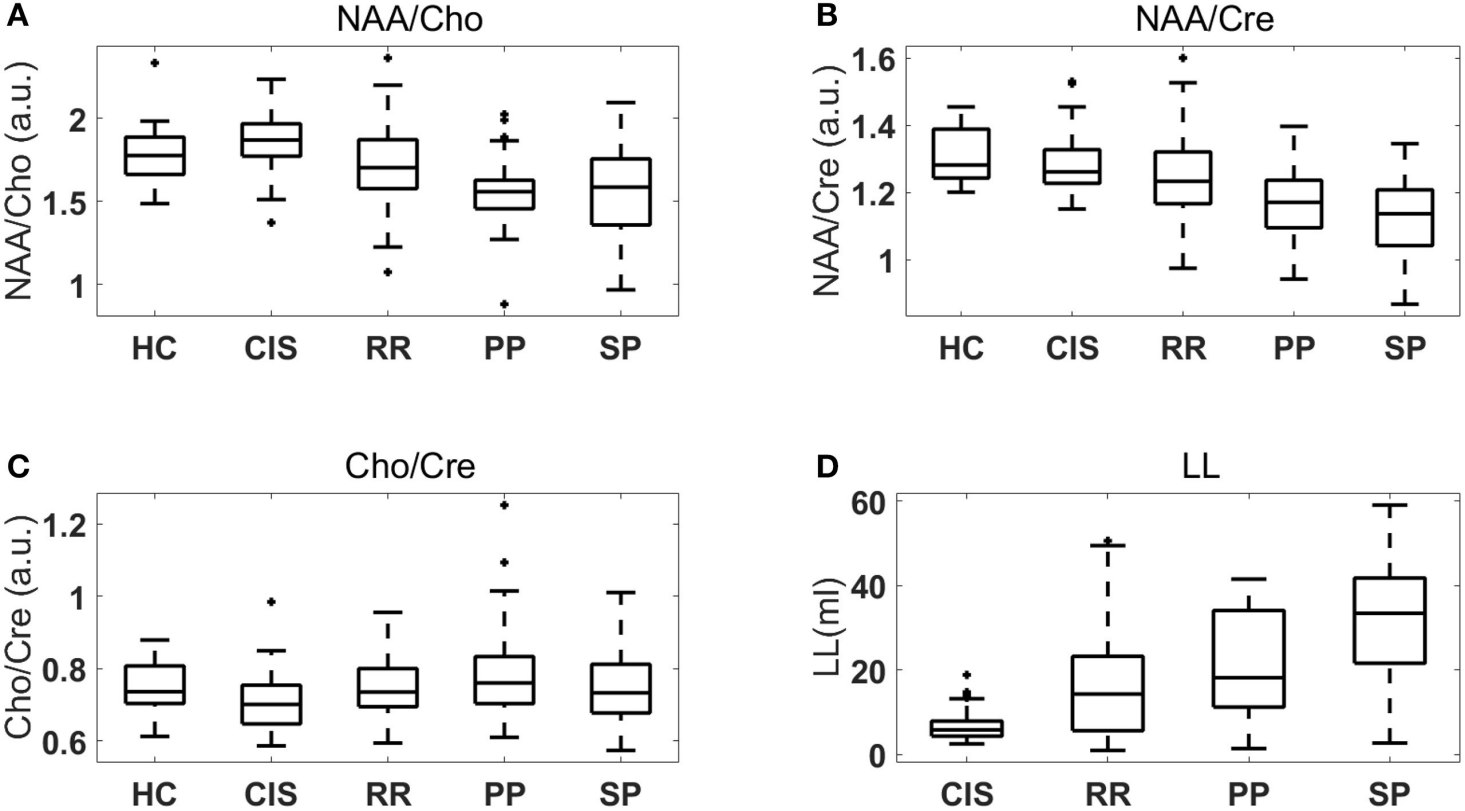
Boxplots of MR metabolic features and lesion loads extracted from HC and MS patients: **(A)** NAA/Cho; **(B)** NAA/Cre; **(C)** Cho/Cre; **(D)** Lesion load (LL).

Figure 1 shows boxplots comparing MR metabolic features (Figures 1A–C) and lesion loads (Figure 1D) extracted from HC and each MS course. Boxplots are drawn using default style in MatLab, meaning the middle line inside the box represents the median value, the vertical limits are the 25th and 75th percentiles (*q*_1_ and *q*_3_), each whisker covers 1.5 the interquartile range (i.e., *q*_3_−*q*_1_), and the crosses outside the whiskers represent outliers. Supplementary Figures 1–4 show the MS data points in various 2-D feature spaces.

Using the previously described (Section 2.6) linear mixed-effects models we found that the fixed effect MS course is statistically significant in the evolution of NAA/Cho, NAA/Cre, Cho/Cre, and LL, with corresponding *p*-values of: 3.4 × 10^−6^, 2 × 10^−4^, 2.3 × 10^−2^, and 2.6 × 10^−4^. Table 3 provides adjusted *p*-values for multiple comparisons between the MS groups.

**Table 3:** Adjusted *p*-values for multiple comparisons between MS groups modeled by linear mixed effects model, tested using the “multcomp” package in “R” (^*^p < 0.05 and ^**^p < 0.01).

	**CIS-RR**	**RR-PP**	**RR-SP**
NAA/Cho	–	^**^	^**^
NAA/Cre	–	–	^*^
Cho/Cre	–	–	–
LL	–	–	^*^

Table 4 shows F1-scores after training LDA using only metabolic ratios, as clinical data and lesion loads were not available for healthy controls. Corresponding BAR, sensitivity and specificity values of this table can be found in Table A1 in Appendix. If F1-scores are missing, then the classifier assigned all data points to the negative class (second MS group).

**Table 4:** F1-scores for all nine classification tasks (rows) after training LDA using only metabolic ratios.

	**NAA/Cho**	**NAA/Cre**	**Cho/Cre**	**All 3 metabolic ratios**
HC vs. CIS	35	33	43	36
HC vs. RR	6	16	–	14
HC vs. PP	47	45	19	49
HC vs. RR+SP	8	19	–	16
HC vs. PP+SP	21	26	–	28
CIS vs. RR	15	–	–	21
CIS vs. RR+SP	3	–	–	19
RR vs. PP	75	78	75	74
RR vs. SP	60	67	58	69

*Values above 75 are colored in light gray*.

Surprisingly, the F1-scores for separating HC from any MS course are very low, and the same holds true for separating very early MS form (CIS) and the most likely MS evolution, RR and RR+SP. In contrast, for RR vs. PP we find that all three metabolic ratios have F1-scores higher than 75, with a maximum of 78 for NAA/Cre. For RR vs. SP the F1-scores are lower, with a maximum of 69 after combining all metabolic features.

Table 5 shows F1-scores of classification tasks involving only MS patients. Training was done on seven different combinations of features to evaluate the classification power of clinical data, lesion loads, and metabolic features. Corresponding BAR, sensitivity, and specificity values can be found in Appendix in Tables A2–A4, respectively. If F1-scores are missing, then the classifier assigned all data points to the negative class (second MS group).

**Table 5:** F1-scores for classification tasks involving only MS patients (columns).

	**CIS vs. RR**			**CIS vs. RR**+**SP**			**RR vs. PP**			**RR vs. SP**		
	**LDA**	**SVM-rbf**	**RF**	**LDA**	**SVM-rbf**	**RF**	**LDA**	**SVM-rbf**	**RF**	**LDA**	**SVM-rbf**	**RF**
M	21	48	11	19	31	–	74	52	73	69	70	67
LL	–	51	27	–	40	24	71	19	73	75	77	68
Age + DD	48	58	51	44	56	50	79	64	74	76	75	71
Age + DD + EDSS	55	65	49	57	66	48	85	81	79	84	85	84
Age + DD + EDSS + LL	67	71	59	63	72	60	79	75	79	86	86	86
Age + DD + EDSS + M	56	59	48	60	59	51	85	83	80	86	87	85
Age + DD + EDSS + LL + M	65	64	57	65	63	57	83	81	78	87	87	86

*M, all three average metabolic ratios; Age, patient age; DD, disease duration; LL, lesion load; EDSS, Expanded Disability Status Scale. Values between 75 and 79 are colored in light gray, values between 80 and 84 are colored in medium gray, while values larger than 85 are colored in dark gray*.

The highest F1-scores for CIS vs. RR and CIS vs. RR+SP, respectively 71 and 72, were achieved by SVM-rbf trained on clinical data and lesion loads. Training any classifier only on metabolic features yielded very low F1-scores.

The highest F1-score for RR vs. PP (85) was achieved by LDA using patient age, disease age, and EDSS. Adding all spectroscopic information maintained the F1-score at 85, while adding lesion load lowered the F1-score at 79. LDA outperformed SVM-rbf and RF in all RR vs. PP cases, always achieving an F1-score higher than 70.

The highest value for RR vs. SP (87) was first achieved after training SVM-rbf on clinical and metabolic features, but also with LDA trained on all features combined (clinical data, lesion loads, and metabolic features). SVM-rbf outperfomed LDA in the majority RR vs. SP cases, but only with 1–2%.

## 4. Discussion

In this paper, we present results for nine binary classification problems using clinical data, lesion loads and metabolic features extracted from MS patients and healthy controls. We focused on metabolic features as numerous studies showed significant metabolic alterations in MS patients of different MS forms. It has been demonstrated that metabolic abnormalities in MS patients are not restricted to lesions alone (Husted et al., 1994; Doyle et al., 1995; Narayanan et al., 1997; Fu et al., 1998; Narayana et al., 1998; Sarchielli et al., 1999; He et al., 2005) and NAWM tissue is well known to be altered in MS (Narayana, 2005; De Stefano and Filippi, 2007). Concentrations of NAA in NAWM were shown to be significantly lower in MS patients (Bitsch et al., 1999; Suhy et al., 2000; Bjartmar et al., 2001; Inglese et al., 2003; Tiberio et al., 2006; Wattjes et al., 2007, 2008). Concentrations of Cho and Cre in NAWM were shown to be significantly higher in MS patients (Narayana et al., 1998; Tourbah et al., 1999; Suhy et al., 2000; Tartaglia et al., 2002; Inglese et al., 2003). Concentrations of NAA/Cre in NAWM were shown to be significantly lower in MS patients (Leary et al., 1999; Narayana et al., 2004). Multiple studies also report significant differences between metabolite concentrations in lesions vs. NAWM of HC: lower NAA and increased Cho and Cre (Wolinsky et al., 1990; Larsson et al., 1991; Davie et al., 1994, 1997; Narayana et al., 1998; Arnold et al., 2000; He et al., 2005).

Our findings are in agreement with these previous reports as decreased NAA and increased Cho and Cre contents were measured in NAWM and lesions of MS patients. After building linear mixed-effects models to properly analyze the statistical difference between the four clinical courses, we observed significant differences at the disease starting points of all MS courses using four response variables, namely the lesion load, NAA/Cre, NAA/Cho, and Cho/Cre ratios. A cross-sectional study (Hannoun et al., 2012) based on a subset of our MRSI data found statistical differences in the NAA/Cre and NAA/Cho ratios between HC and RR, PP, SP, and RR+PP+SP patients. To our knowledge, there is only one study that reports sensitivity and specificity values for classifying healthy controls from MS patients based on spectroscopic features. Inglese et al. (2003) show that absolute values of choline in NAWM can differentiate 9 controls and 10 out of 11 RR patients.

Other MS classification studies are Muthuraman et al. (2016) and Kocevar et al. (2016), both based on diffusion features. The first one reports a classification accuracy of 97% between 20 CIS and 33 RR patients. The second one analyzes classification tasks based on DTI data from a cross-sectional subset of our database. They found very high F1-scores (91.8% for both HC-CIS and CIS-RR) after training SVM-rbf on six global brain connectivity metrics. For RR vs. PP their maximum F1-score was 75.6%, which is lower than our results based on metabolic features, while for RR vs. SP, their maximum F1-score was 85.5%, which is comparable to our results. It is also worth mentioning that they did not use any clinical data, which might improve their results.

In this study, we analyzed the added value of combining standard clinical data with quantitative magnetic resonance features. To this purpose, we trained linear and non-linear classifiers only on advanced MR features, and then only on clinical data. Afterwards we train the classifiers on clinical data combined with lesion loads and metabolic features.

Although, MS patients are expected to have significantly different WM metabolism compared to healthy controls, this difference was not reflected in the metabolic average obtained from “good quality” voxels (Supplementary Figures 1A,B). This result is not entirely surprising, considering that we averaged over a high number of voxels, and the subtle lesion information could be lost in the average. However, we can visually see in Supplementary Figures 1C,D that the two progressive MS courses tend to have lower NAA/Cho and NAA/Cre ratios than healthy controls.

CIS and RR patients' distribution over the NAA/Cho and NAA/Cre feature space do not differ much, as seen in Supplementary Figure 2A. Disease duration interval for RR patients is much larger than for CIS patients, as most of CIS patients have a disease duration lower than 5 years, which can be seen in Supplementary Figure 3A. Because RR patients have more relapses than CIS patients, the number of lesions will be higher and the lesion volume as well, while EDSS scores are mainly in the same range, as seen in Supplementary Figure 4A. BAR values in Table A2 show a maximum of 85, when combining patient age, disease duration, EDSS, and lesion load. However, the corresponding maximum F1-score of 71 is much lower because the dataset is unbalanced (61 CIS vs. 214 RR), heavily influencing the classifier's precision. In this case the F1-score reflects better than BAR the difficulty of discriminating CIS from RR forms.

CIS and SP patients' distribution over different features is visible in Supplementary Figures 2B, 3B, 4B and it is clear that these two are the least and most advanced forms of MS. Because RR patients will eventually evolve into SP forms during their lifetime, we grouped together RR and SP patients for a separate classification task versus CIS patients. BAR values in Table A2 show a maximum of 92, when combining patient age, disease duration, EDSS and lesion load. The same discussion as for CIS vs. RR apply: the corresponding maximum F1-score is only 72 because the dataset is very unbalanced (61 CIS vs. 410 RR+SP) and the precision will be very low.

RR and PP patients can be discriminated using only EDSS by visually inspecting Supplementary Figure 4C. Training a linear classifier on clinical data (patient age, disease duration, and EDSS) gives the maximum F1-score of 85. Adding the 3 metabolic features keeps the score at 85, while adding lesion load information lowers the score to 79. This drop in the F1-score suggests that lesion load is not useful in differentiating RR from PP patients. Indeed, these two MS forms have the closest lesion load averages (16.7 and 20.8 ml), as shown in Table 1. In contrast, the clinical status of RR and PP patients are very different, as reflected by the EDSS values of 2 for RR and 4 for PP. Moreover, training LDA on individual metabolic features always provided higher F1-scores than lesion load, therefore we can conclude that for RR vs. PP, metabolic features have a higher discrimination power than LL. BAR values in Table A2 are also closer to the F1-scores in Table 5 because the dataset is more balanced compared to previous cases.

RR and SP patients can also be discriminated using only EDSS by visually inspecting Supplementary Figure 4D. Our results showed that EDSS is very important in differentiating RR patients from primary or secondary progressive patients. We also report consistent higher F1-scores for classifiers trained only on lesion load compared to classifiers trained only on metabolic features. Furthermore, it is clearly visible in Table 4 that we obtain higher F1-scores for this classification task using multiple features, compared to the rest of 8 tasks. These findings suggest that in the future it might be possible to build a decision support system using clinical data combined with lesion loads and metabolic features.

However, this study suffers from a few limitations caused by the low scanning frequency of only 1.5 Tesla. Firstly, it is known that the sensitivity of lesion load segmentation is improved by scanning at higher frequencies (Sicotte et al., 2003). Therefore, our LL values may not reflect entirely the pathological changes inside the brain. Secondly, the signal to noise ratio of MRSI is proportional to the scanning frequency, meaning our metabolites' quantification is not entirely accurate. In order to obtain true metabolites values, we would have to measure T1 and T2 relaxation times of water for each patient, which would be impossible in clinical practice. Moreover, spectroscopic signal scales can differ from patient to patient, resulting in large metabolite variations. To overcome some of these limitations, we use as features all three metabolite ratios (NAA/Cho, NAA/Cre, Cho/Cre). By doing so, we expect to retain the most valuable information.

When comparing classification tasks from a computational point of view, LDA is clearly the winner as the training period last only 3 h using a computer with 8 threads. Training both SVM-rbf and RF took around 20 days in total and it was done using 60 threads, meaning LDA is approximately 600 times faster than SVM-rbf or RF. Also, the maximum F1-scores for RR vs. PP and RR vs. SP were obtained with LDA and SVM-rbf, suggesting that a linear classifier performs equally good as a non-linear classifier in these cases.

This study is a proof of concept that investigates the added value of MR metabolites combined with clinical data and lesion loads, in classifying MS patients and healthy controls. Clinical data is routinely collected by doctors, lesion load is a known marker of neurodegeneration, while MR metabolites have been shown to provide high specificity of MS pathology. In order to better understand the underlying MS pathological mechanisms, we used three different machine learning methods, one linear and two non-linear, and had a strict quality control for extracting metabolic features. Despite all our efforts, averaging metabolite ratios over “good quality” voxels provides only moderate biomarkers for discriminating MS groups (i.e., RR vs. PP). In general, combining patient age, disease duration, EDSS, and averaged metabolic ratios, leads to the highest classification results. We believe extracting metabolic information from specific brain sub-regions of the MRSI grid (e.g., NAWM) should provide a more detailed view of MS pathology and help the classification tasks. Therefore, further investigations about the MS patients' evolution will be done in the future on sub-regions metabolite quantification, DTI-based brain connectivity metrics, patient treatment, and multi-class classification.

## 5. Conclusions

In this paper, we performed nine binary classification tasks and report F1-scores and BAR values after learning linear and non-linear classifiers on combinations of clinical data, lesion loads, and metabolic features. We presented a simple method to compute metabolic features by averaging metabolite ratios over “good quality” voxels of a MRSI grid. Using linear mixed-effects models we found that the MS course is statistically significant in the evolution of four response variables: Lesion Load, NAA/Cre, NAA/Cho, and Cho/Cre ratios. Our results showed that the best classifier for discriminating CIS from RR or RR+SP is SVM-rbf trained on clinical data and lesion loads. We also showed that discriminating RR from PP or SP with high accuracy is possible when training LDA on clinical data. For RR vs. PP, adding metabolic features will not change the results, while for RR vs. SP, adding metabolic features and lesion loads will slightly improve the results.

## Author contributions

AI, GK, CS, DS, SV, and DS designed the methodological part of the study. FD and DS designed the clinical part of the study and collected the data. AI analyzed the data. All authors contributed to the manuscript.

### Conflict of interest statement

The authors declare that the research was conducted in the absence of any commercial or financial relationships that could be construed as a potential conflict of interest.

## Supplementary material

Supplementary Figure 1 **(A–D)** HC vs. MS groups: NAA/Cho vs. NAA/Cre.

Supplementary Figure 2 **(A–D)** MS groups comparison: NAA/Cho vs. NAA/Cre.

Supplementary Figure 3 **(A–D)** MS groups comparison: Disease age vs. Cho/Cre.

Supplementary Figure 4 **(A–D)** MS groups comparison: Lesion Load vs. EDSS.

## A. Appendix

**Table A1:** Balanced accurary rates (BAR), sensitivity (SEN), and specificity (SPE) values, for all 9 classification tasks (rows) after training LDA using only metabolic ratios.

	**NAA/Cho**			**NAA/Cre**			**Cho/Cre**			**All 3 metabolites**		
	**BAR**	**SPE**	**SEN**	**BAR**	**SPE**	**SEN**	**BAR**	**SPE**	**SEN**	**BAR**	**SPE**	**SEN**
HC vs. CIS	47	0	94	46	15	78	61	39	83	53	39	67
HC vs. RR	50	94	6	55	82	28	50	100	0	52	76	28
HC vs. PP	76	80	72	78	72	83	45	29	61	77	82	72
HC vs. RR + SP	52	98	6	60	92	28	50	100	0	59	90	28
HC vs. RR + PP	61	89	33	66	88	44	50	100	0	52	88	16
CIS vs. RR	52	95	10	50	100	0	50	99	0	52	88	16
CIS vs. RR + SP	51	100	2	49	99	0	50	100	0	54	94	15
RR vs. PP	59	37	81	63	38	88	48	2	95	63	49	77
RR vs. SP	57	53	62	65	62	69	39	0	79	66	62	70

*Values between 75 and 79 are colored in light gray, values between 80 and 84 are colored in medium gray, values between 85 and 89 are colored in dark gray, while values higher than 90 are colored in very dark gray*.

**Table A2:** BAR values for classification tasks involving only MS patients (columns).

	**CIS vs. RR**			**CIS vs. RR**+**SP**			**RR vs. PP**			**RR vs. SP**		
	**LDA**	**SVM-rbf**	**RF**	**LDA**	**SVM-rbf**	**RF**	**LDA**	**SVM-rbf**	**RF**	**LDA**	**SVM-rbf**	**RF**
M	52	68	49	54	63	49	63	28	59	66	66	63
LL	48	70	52	50	73	56	43	12	58	74	75	68
Age + DD	66	75	68	66	83	70	67	38	62	75	76	71
Age + DD + EDSS	71	80	67	77	89	69	81	78	70	84	85	84
Age + DD + EDSS + LL	79	85	73	81	92	76	71	72	69	86	86	85
Age + DD + EDSS + M	72	76	66	81	82	70	80	81	71	86	87	84
Age + DD + EDSS + LL + M	78	80	71	82	83	73	78	78	68	86	86	86

*M, all three average metabolic ratios; Age, patient age; DD, disease duration; LL, lesion load; EDSS, Expanded Disability Status Scale.Values between 75 and 79 are colored in light gray, values between 80 and 84 are colored in medium gray, values between 85 and 89 are colored in dark gray, while values higher than or equal to 90 are colored in very dark gray*.

**Table A3:** Sensitivity values for classification tasks involving only MS patients (columns).

	**CIS vs. RR**			**CIS vs. RR**+**SP**			**RR vs. PP**			**RR vs. SP**		
	**LDA**	**SVM-rbf**	**RF**	**LDA**	**SVM-rbf**	**RF**	**LDA**	**SVM-rbf**	**RF**	**LDA**	**SVM-rbf**	**RF**
M	16	79	8	15	67	0	77	56	78	70	75	72
LL	0	80	30	0	80	23	87	16	78	77	80	67
Age + DD	41	77	49	36	84	46	84	75	78	74	70	70
Age + DD + EDSS	56	82	44	62	92	43	84	75	80	80	83	81
Age + DD + EDSS + LL	69	87	56	69	93	57	81	69	83	85	84	85
Age + DD + EDSS + M	59	74	41	74	79	44	84	76	82	84	85	84
Age + DD + EDSS + LL + M	67	79	49	72	77	51	83	75	81	87	87	86

*M, all three average metabolic ratios; Age, patient age; DD, disease duration; LL, lesion load; EDSS, Expanded Disability Status Scale. Values between 75 and 79 are colored in light gray, values between 80 and 84 are colored in medium gray, values between 85 and 89 are colored in dark gray, while values higher than or equal to 90 are colored in very dark gray*.

**Table A4:** Specificity values for classification tasks involving only MS patients (columns).

	**CIS vs. RR**			**CIS vs. RR**+**SP**			**RR vs. PP**			**RR vs. SP**		
	**LDA**	**SVM-rbf**	**RF**	**LDA**	**SVM-rbf**	**RF**	**LDA**	**SVM-rbf**	**RF**	**LDA**	**SVM-rbf**	**RF**
M	88	57	89	94	59	98	49	0	40	62	56	54
LL	96	60	75	100	66	89	0	7	37	70	70	69
Age + DD	91	74	87	96	83	94	50	0	46	76	82	72
Age + DD + EDSS	87	79	89	91	87	95	78	81	60	89	87	86
Age + DD + EDSS + LL	89	83	90	92	90	95	60	75	55	87	87	85
Age + DD + EDSS + M	85	78	91	89	86	95	75	86	60	88	88	84
Age + DD + EDSS + LL + M	88	81	93	92	89	96	74	82	56	85	86	85

*M, all three average metabolic ratios; Age, patient age; DD, disease duration; LL, lesion load; EDSS, Expanded Disability Status Scale. Values between 75 and 79 are colored in light gray, values between 80 and 84 are colored in medium gray, values between 85 and 89 are colored in dark gray, while values higher than or equal to 90 are colored in very dark gray*.
