# Supplementary figures and images for: Machine Learning Approach for Classifying Multiple Sclerosis Courses by Combining Clinical Data with Lesion Loads and Magnetic Resonance Metabolic Features
Source: Front Neurosci. 2017 Jul 11;11:398. doi: 10.3389/fnins.2017.00398 (PMC5504183; doi:10.3389/fnins.2017.00398)

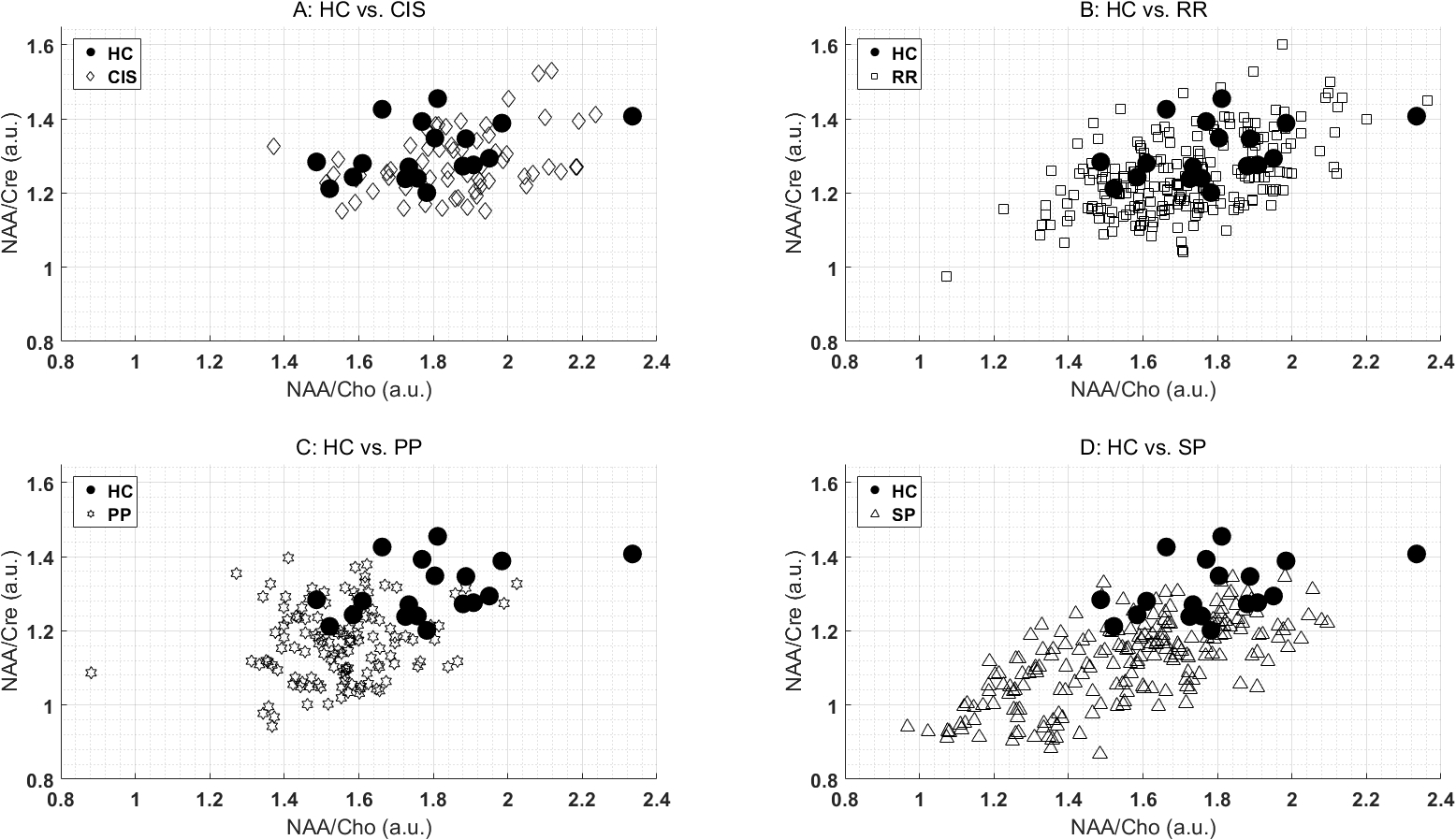

Supplement: Supplementary Figure 1 — (A–D) HC vs. MS groups: NAA/Cho vs. NAA/Cre. [file Image1.jpeg]

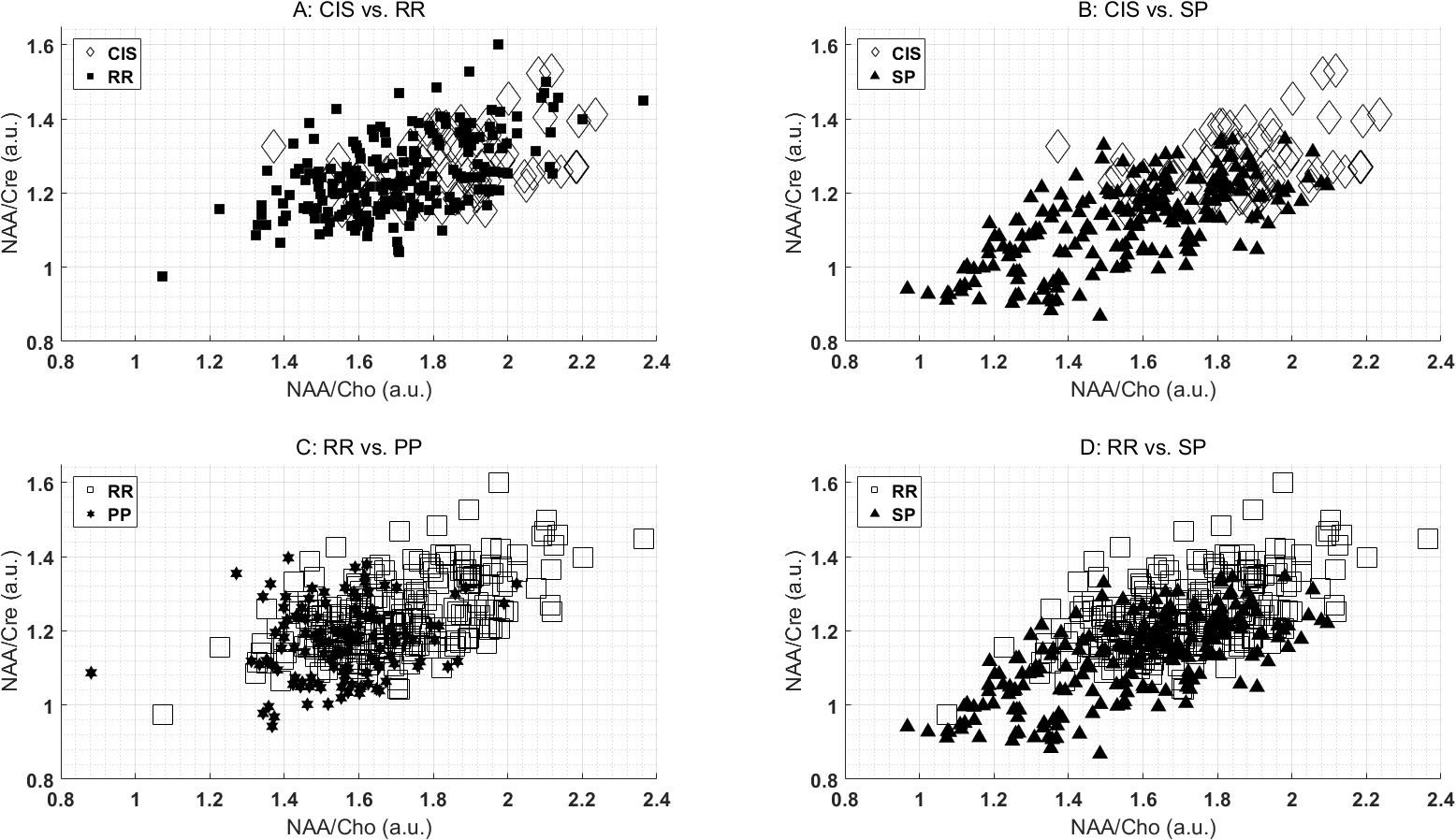

Supplement: Supplementary Figure 2 — (A–D) MS groups comparison: NAA/Cho vs. NAA/Cre. [file Image2.jpeg]

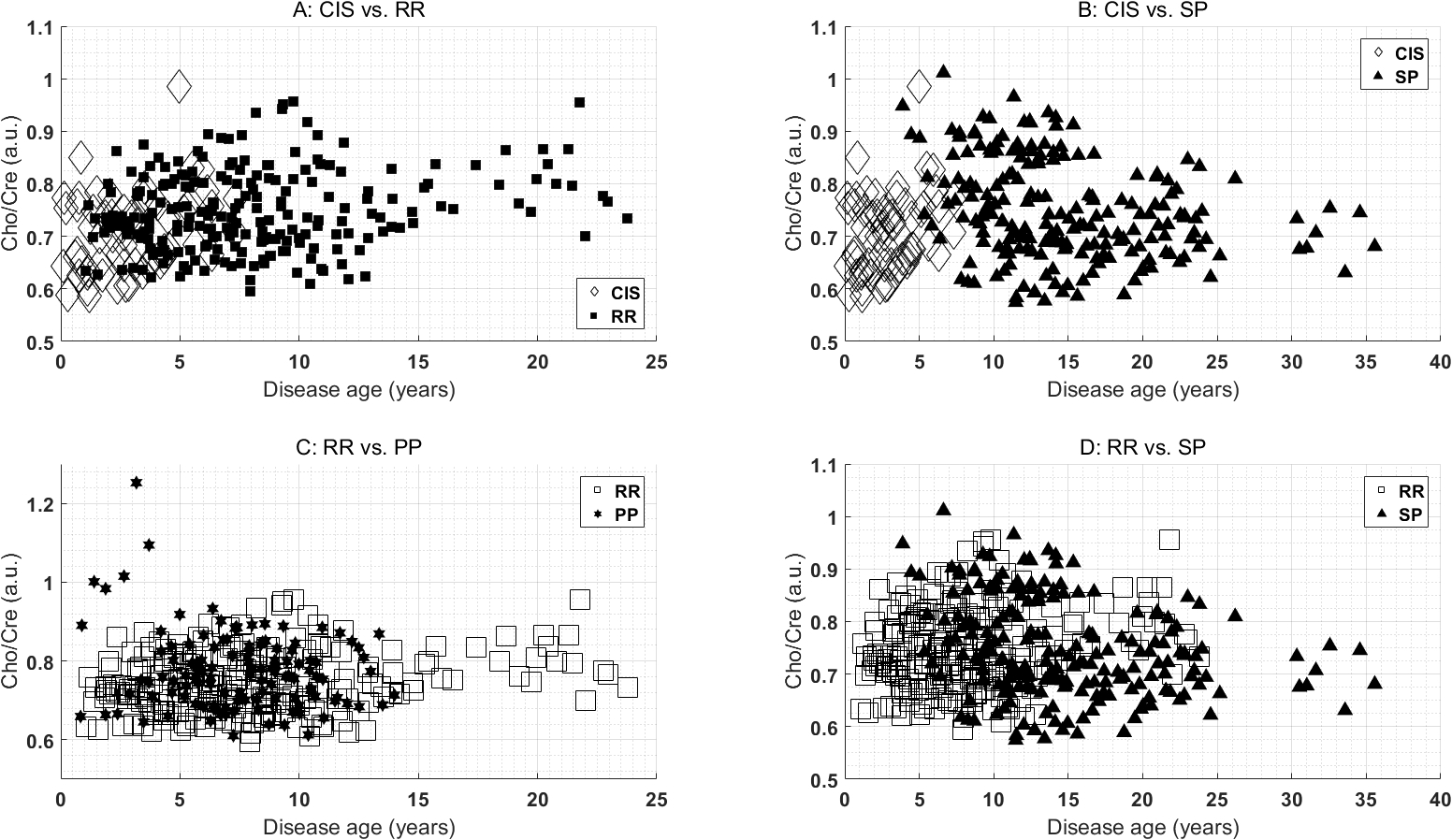

Supplement: Supplementary Figure 3 — (A–D) MS groups comparison: Disease age vs. Cho/Cre. [file Image3.jpeg]

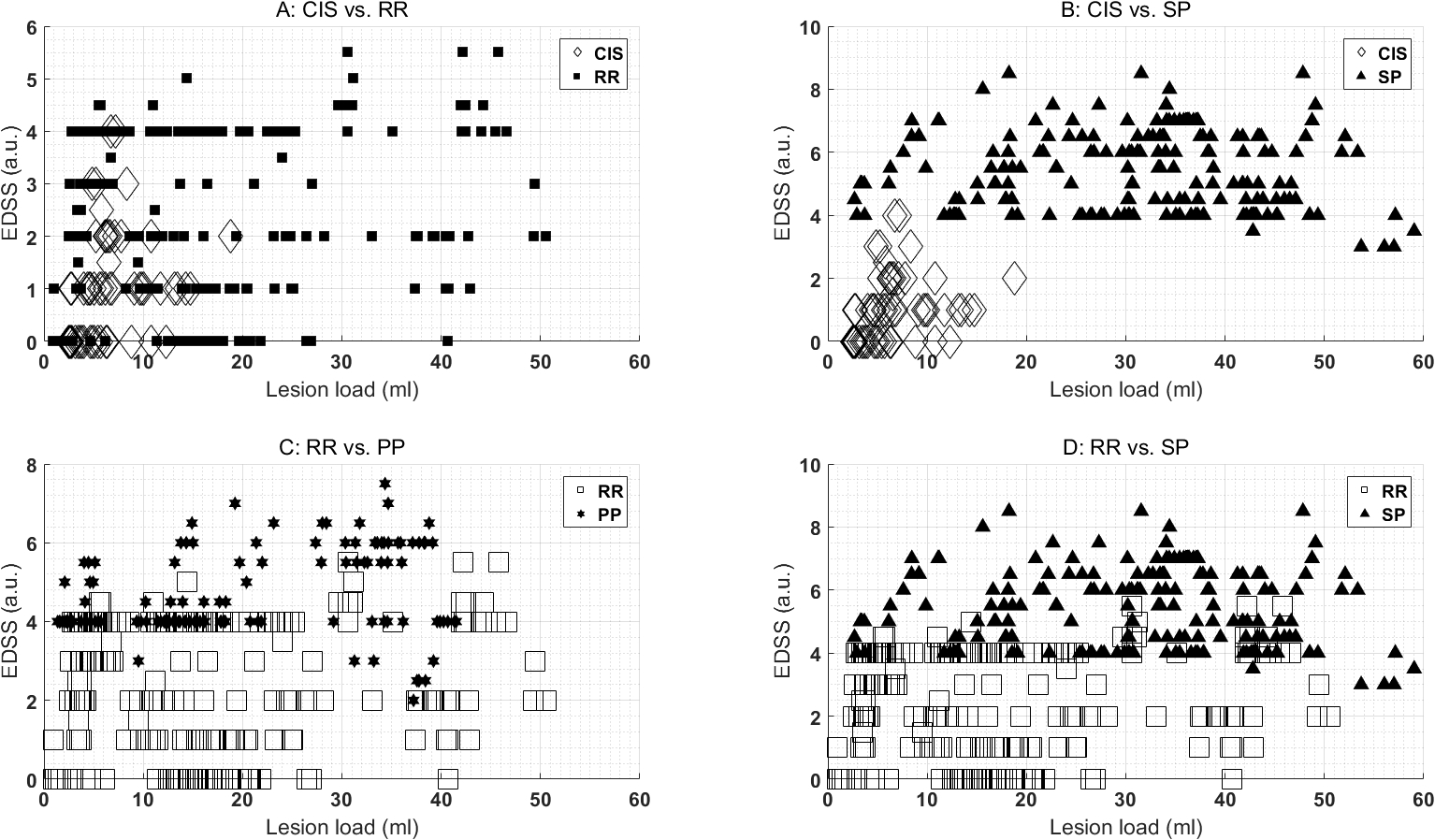

Supplement: Supplementary Figure 4 — (A–D) MS groups comparison: Lesion Load vs. EDSS. [file Image4.jpeg]
